# *Bandavirus dabieense*: A review of epidemiology, clinical characteristics, pathophysiology, treatment and prevention

**DOI:** 10.1080/21505594.2025.2520343

**Published:** 2025-06-16

**Authors:** Chengcheng Peng, Yujia Hao, Yuge Yuan, Wenzhou Ma, Duo Zhang, Jienan Kong, He Zhang, Nan Li, Pengpeng Xiao

**Affiliations:** a Wenzhou Key Laboratory for Virology and Immunology, Institute of Virology, Wenzhou University, Wenzhou, China; b Department of Pathology, First Affiliated Hospital of Dalian Medical University, Dalian, China; c Changchun Veterinary Research Institute, Chinese Academy of Agricultural Sciences, Changchun, China

**Keywords:** Bandavirus dabieense, epidemiology, diagnosis, prevention, treatment

## Abstract

Bandavirus dabieense (commonly known as severe fever with thrombocytopenia syndrome virus, SFTSV) infection leads to severe fever with thrombocytopenia syndrome (SFTS), which is an emerging tick-borne natural focus disease discovered in middle-eastern China. SFTS is characterized by fever with thrombocytopenia, and patients’ main clinical manifestations are leucopenia, elevated serum liver enzymes, and multiple organ failure. Ticks are considered as carriers of SFTSV transmission, and *Haemaphysalis longicornalis* is considered the main vector tick. SFTSV is disseminated through the migration or movement of tick-carrying migratory birds and other animal hosts. With changes in climate and environment, the habitat of ticks such as *haemaphysalis longicornalis* are continuously expanding, coupled with the diverse animal host species of the ticks. SFTS is evolving into a serious global public safety issue. In the absence of specific treatments and vaccines still being developed, monitoring and vector control are crucial for curbing the spread of SFTSV. Here, based on the existing literature, we reviewed the epidemiology, infection mechanism, clinical characteristics, diagnosis, prevention and clinical treatment of SFTSV to enhance the understanding of the SFTSV, with the aim of providing a theoretical basis guidance for the government and relevant institutions to prevent and control the further spread of SFTSV.

## Introduction

Bandavirus dabieense (commonly known as severe fever with thrombocytopenia syndrome virus, SFTSV) is a tick-borne virus of the genus *Bandavirus*, belongs to the family *Phenuiviridae* of the order *Bunyavirales* [1]. SFTSV infection can cause SFTS, with clinical manifestations of acute high fever, thrombocytopenia, leucopenia, elevated serum liver enzymes, gastrointestinal symptoms, and multiple organ failure [2]. In the case of SFTS, it can also induce or aggravate complications of patients, such as abnormal lymph function, diabetes, liver and kidney function. The combined effect of multiple diseases leads to differences in the mortality rate of SFTS patients, with an average mortality rate of 12% and even up to 30% in some regions [3]. In recent years, the SFTSV epidemics have gradually expanded, posing a great threat to human life and health. SFTS is listed among the top ten priority infectious diseases by World Health Organization [4].

**Figure 1.**
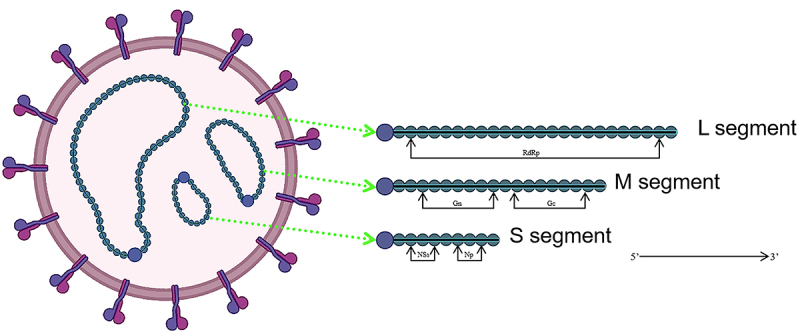
Genomic structure of SFTSV. SFTSV is an enveloped, segmentalized negative-stranded RNA virus, with spherical virions about 80 ~ 100 nm in diameter. The SFTSV genome consists of three RNA segments, large (L), medium (M), and small (S). The L segment encodes the RNA-dependent RNA polymerase (RdRp) and the M segment encodes the Gp, which can be cut and processed by cellular proteases into two subunits: glycoprotein N (Gn) and glycoprotein C (Gc) during translation. The S fragment belongs to the ambisense RNA family and has two opposite reading frames encoding two proteins from both directions, the 3’end reverse sequence encoding nuclear protein (NP) and the 5’ end sequence encoding non-structured (NSs) protein.

SFTSV was first isolated in 2009 from patients with fever, thrombocytopenia, leucopenia and multi-organ dysfunction in Hubei and Henan provinces of China. Since its discovery, SFTSV has been named several times by the International Committee on Taxonomy of Viruses (ICTV): the SFTS virus (2014), the SFTS phlebovirus (2015), the Huaiyangshan banyangvirus (2018), and the Dabie bandavirus (2020). SFTSV is an enveloped, segmentalized negative-stranded RNA virus, with spherical virions about 80 ~ 100 nm in diameter [5] (Figure 1). The SFTSV genome consists of three RNA segments, large (L), medium (M), and small (S), whose end sequences are highly conserved to form a narrow stem-like structure, which is enclosed by a surface envelope consisting of two transmembrane glycoproteins and a lipid bilayer with spinous processes, showing icosahedral symmetry [6,7]. The three segments of the 11.5 kb viral genome undertake different coding tasks. The L segment encodes the RNA-dependent RNA polymerase (RdRp) and the M segment encodes the Gp, which can be cut and processed by cellular proteases into two subunits: glycoprotein N (Gn) and glycoprotein C (Gc) during translation. The S fragment belongs to the ambisense RNA family and has two opposite reading frames encoding two proteins from both directions, the 3 “end reverse sequence encoding nuclear protein (NP) and the 5” end sequence encoding non-structured (NSs) protein [8]. As with other segmented negative chain viruses (sNSVs), SFTSV is released into the host cytoplasm and each segment transcribes mRNA separately, which is then translated into the corresponding viral protein [9].

SFTSV has wide distribution of vectors and variety of animal hosts, which has become a major public health issue [10,11]. However, there is currently no specific drug or vaccine for SFTSV, and the pathogenicity and lethal mechanism of SFTSV still need to be studied in depth [12,13]. Due to its high fatality rate and the potential for pandemic spread, SFTSV has recently been designated as a priority pathogen by the World Health Organization (WHO) and the National Institute of Allergy and Infectious Diseases (NIAID) of the United States [14]. Thus, here we review recent investigations of SFTSV, including the epidemiology of SFTSV, infection mechanism, clinical characteristics, diagnosis, prevention and clinical treatment of SFTSV.

## Methods

A comprehensive search was conducted across three databases: Pubmed, Web of Science, and CNKI. To ensure a thorough search, we used the search terms “Bandavirus dabieense” and “severe fever with thrombocytopenia syndrome virus” within the Title/Abstract fields of these databases. In addition, the global distribution data *Haemaphysalis longicornis* detected between 1978 ~ 2023 were obtained based on the records of GBIF.org

## Epidemiology

### Geographical distribution

Reported cases of SFTS are mainly in the Asian region, especially in East Asia, where the incidence rate is higher. SFTS was first reported in the Henan province in central China in 2009, and quickly spread to Zhejiang, Jiangsu, Shandong and other provinces. Currently, SFTSV infection events have been reported in 27 provinces in China [15]. Subsequently, patients with SFTS have been reported in the United States, Japan, Thailand, Vietnam, and South Korea, and none of these patients have travelled abroad, suggesting that SFTS occurs with some natural epidemic origin [16–18].

### Reservoirs and vectors

SFTSV is transmitted through tick bites, and a study of the carrying time of SFTSV infection in different tick species found that *Ixodes persulcatus* and *Dermacentor silvarum* carried SFTSV for 9 and 6 days, respectively, while *Haemaphysalis longicornis* carried it for up to 21 days [19]. *Haemaphysalis longicornis* can serve as a vector capable of transovarial and transstadial transmitting SFTSV. Duration of SFTSV-carrying days in adults of *Haemaphysalis longicornis* laid the foundation of transmission of SFTSV. The *Haemaphysalis longicornis* is considered the primary vector of SFTSV with a wide host range [20]. *Haemaphysalis longicornis* are rare populations with both sexual and parthenogenetic reproduction [21].

Since SFTSV is transmitted in *Haemaphysalis longicornis* by two routes: transovarial and transstadial transmission, SFTSV can circulate throughout the life cycle of *Haemaphysalis longicornis* [22]. Thus, the *Haemaphysalis longicornis* is not only a major vector of SFTSV, but also an important reservoir host for SFTSV. Throughout the tick’s development, from larvae, to warts, to adults, each stage requires blood-sucking to change hosts [23]. SFTSV-infected ticks attach to the skin of the host to suck blood and release the virus into the host. Moreover, ticks can also be infected with SFTSV from different hosts during the blood-sucking process, which allows for a persistent cycle of SFTSV transmission in nature.

**Figure 2.**
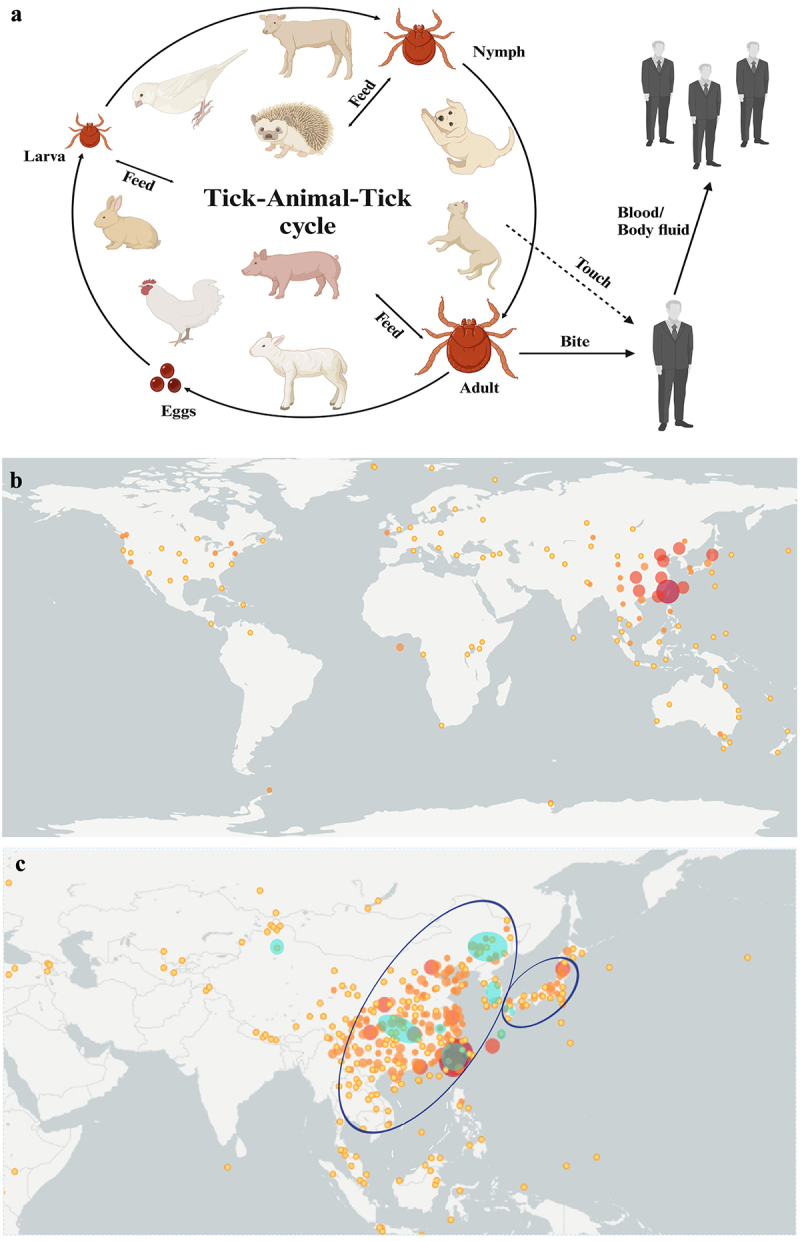
Spread of SFTSV. (a), Transmission of SFTSV among ticks, animals and humans. SFTSV can be transmitted throughout the entire development cycle of *Haemaphysalis longicornis*, from larva, warts to adults, requiring a bloodsucking host change to form the tick-vertebrate-tick cycle. Humans are infected with SFTSV primarily through tick bites or through direct contact with the blood and/or body fluids of infected animals or patients (Created with BioRender.com). (b), the global distribution data *Haemaphysalis longicornis* detected between 1978 ~ 2023, the database comes from GBIF.Org (June 7, 2023) GBIFOC currence. Download at https://doi.org/10.15468/dl.Tvwagv, screening coordinate instrument uncertainty value <3. The size of the circle represents the abundance of *Haemaphysalis longicornis*. (c), Distribution of SFTS cases and positive animal samples in the Asian region. The green solid circle represents the distribution of SFTS cases, mainly concentrated in the southeastern coastal areas of China, japan, and South Korea. The blue hollow circle represents the animal samples that have detected SFTSV, including other ticks.

Humans can get infected with SFTSV and fall ill through tick bites, and the common hosts of ticks cover most mammals, including companion animals, domestic animals and wildlife. SFTSV can circulate between ticks and vertebrates in a tick-animal-tick cycle [24]. Currently, SFTSV RNA or anti-SFTSV antibodies have been detected in a variety of animals, e.g. sheep, cattle, pigs, dogs, cats, chickens, rodents, wild boars, hedgehogs, raccoons, etc [25–29](Figure 2a). This indicates that SFTSV has a high zoonotic transmission potential.

The *Haemaphysalis longicornis* is widely distributed in East Asia, Australia, New Zealand, and the Hawaiian Islands (Figure 2b). Recently, the tick has invaded the continental United States as an exotic species, possibly through livestock transportation and seasonal migration of migratory birds [30,31]. Migratory birds have long been long-distance carriers of ticks, and it has been found that SFTSV may be transmitted by migratory East Asian birds carrying the *Haemaphysalis longicornis* [30]. These results suggest that migratory birds may contribute to the cross-regional spread of SFTS, implying that SFTSV and other tick-borne pathogens can be transmitted across continents and remain endemic on all continents [32,33]. With global warming and the globalization of trade, SFTSV remains at risk of global transmission [34].

### Seasonal and demographic patterns

The typical season for SFTSV infection is early spring to late fall, and the high prevalence of the disease is among people living and producing in forests, mountains and hilly areas, which is roughly the same as the main habitat of ticks [35]. Tick growth and reproduction are susceptible to climatic factors such as light, humidity and temperature. Higher temperatures and humidity favour the promotion of egg laying by female ticks and the growth and development of young ticks. Seasonal variations in these factors lead to a natural rise and fall in tick densities, resulting in seasonal onset of SFTS in the population [36,37]. The above evidence suggests that changes in environmental factors, especially climatic ecological factors and geomorphic landscape factors, may have provided suitable ecological environments for tick growth and reproduction. In addition, the transportation or migration of tick-carrying hosts across regions has accelerated the increase in tick density and spatial expansion [38].

SFTSV is generally susceptible to the general population. The age range of SFTS cases is from 2 months old to 100 years old, and the high-risk population is the middle-aged and elderly people, and the occupations of the cases are mainly farmers (accounting for generally more than 80%) [39]. In addition, most fatal cases of SFTS occur in patients over 60 years of age, and the mortality rate of SFTS patients increases with age, making advanced age a risk factor associated with disease severity and mortality [40].

### Evolution

SFTSV can evolve in infected ticks and other hosts through genetic mutations and recombination [41]. In the early stages of SFTSV research, sequences from Japan, Korea, and Zhejiang Province, China, have not been reported, and there is insufficient data on SFTSV genome sequences; SFTSV has been categorized into three lineages, and some researchers have also categorized SFTSV into five genotypes [42,43]. After the publication of SFTSV sequences in Japan, Korea and Zhejiang Province of China, SFTSV showed wider genetic diversity, and the six genotypes (A-F) delineation method is now widely used [44–47]. Research has found that three out of six genotypes (F, A, D) are dominant in China, while the B genotype is dominant in South Korea and Japan [48]. The ferret infection studies confirmed that the B genotype has the highest incidence and the A genotype has the lowest mortality, suggesting that SFTSV pathogenicity is related to genotype [49].

SFTS pathogenesis and mortality are directly related to the large number of genomic variants in SFTSV. Studies have shown that the SFTSV S segment has the highest substitution rate and the L segment has the lowest [50]. There are three common variants of SFTSV: Mode I (NS_I233V, Gn_Q341P, and Gn_Q394H), Mode II (NS_S207P, NS_Q245H, Gc_V587I, Gc_T960I, RdRp_T1433A, and RdRp_R1684K), and Mode III (Gn_L337M, RdRp_E397D, RdRp_L703F, GN_L337m, and RdRp_K1825R). Modes I and II are associated with an increased risk of death [51]. It has also been shown that R624W and R962S in the SFTSV glycoprotein precursor (GP) may affect virus-cell fusion [52,53]. N1891K in RdRp may play an important role in polymerase activity [54. P102]A and K211R in nonstructural (NS) proteins inhibit tumour progression site 2-mediated IL-10 production, thereby reducing mortality in SFTSV-infected mice [55].

### Transmission routes

Although humans are primarily infected through tick bites, close contact with companion animals such as cats and dogs and domestic animals increases the risk of human infection. By searching the reports of SFTSV patients and various animal positive samples, we compared their distribution areas, there were overlapping areas, and they were mainly distributed in Japan, South Korea and southeastern provinces of China in Asia (Figure 2c). This can also reflect the relationship between SFTSV patients and animals carrying SFTSV. Therefore, when preventing and controlling SFTSV, attention should be paid not only to ticks, the transmission vector, but also to the surveillance of various animal hosts.

In addition, with the increase in SFTS cases and the deepening of research, it has been found that humans may also be infected through direct contact with blood or body fluids from infected animals or patients [56–61]and studies have shown that SFTSV may be sexually transmitted and *via* aerosol [62–64] (Figure 2a). In recent years, there have been multiple reports that medical staff and family members have been infected in clusters due to direct blood contact with SFTS patients without wearing protective equipment [65]. Thus, Medical staff and family members of patients with SFTS should follow universal precautions when taking care of SFTS patients.

## Mechanisms/Pathophysiology

### Mechanisms of virus intrusion

**Figure 3.**
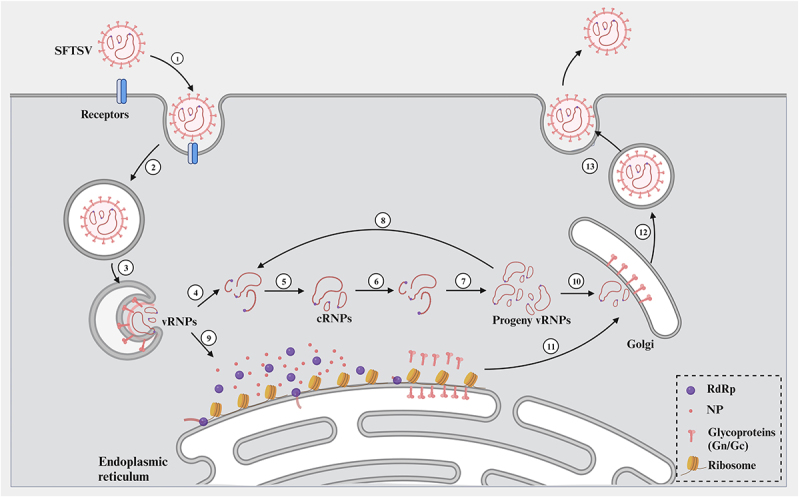
SFTSV replication. ① SFTSV is attached to cells by Gp binding to host DC-SIGN, HS, NMMHC-IIA and other cytokines. ② it is further internalized through the endocytic pathway in a clathrin-dependent manner. ③ in the endosome stage, low pH triggers membrane fusion activity of Gc glycoproteins, allowing viral ribonucleoprotein complexes (vRnps) to be released into the cytoplasm. ④-⑧ vRNA in vRnps directs the synthesis of cRNA, which is assembled with newly synthesized RdRp and NP into complementary RNA (cRnps). Progeny vRnps are generated using cRnps as a template, and progeny vRnps can be used as a template to generate more vRnps. ⑨vrnps released into the cytoplasm migrate to the endoplasmic reticulum, where they direct the synthesis of viral proteins through transcription-coupled translation mechanisms. Viral nucleoproteins and RdRp enzymes are synthesized in the cytoplasm to form ribonucleoprotein (RNP) complexes. The viral glycoprotein GP is translated into the precursor protein Gn/Gc in the rough endoplasmic reticulum (ER). ⑩-⑪ Assembly of virus particles. Properly folded Gn/Gc heterodimers are transported to the golgi organ, where they bind to RNPs via the cytoplasmic tail of Gn during budding. ⑫-⑬ Vesicles containing the virus are transported to the cell membrane, where the virus particles are released through exocytosis (Created with BioRender.com).

Binding of viruses to host cell surface molecules is the primary link in their infection of cells. The entry of Bunyavirales into cells is mainly achieved through receptor-mediated endocytosis and pH-dependent membrane fusion processes [66]. SFTSV entry is a clathrin – and dynamin dependent endocytosis, after which virions are transported to the early endosome and then to the late endosome. In addition, membrane fusion occurs in late endosomes triggered by acidic environments [67] (Figure 3). Liu et al. systematically analysed the dynamic molecular processes of SFTSV entry and penetration by using quantum dot (QD)-based single-particle tracking and multicolour imaging, and verified again that the entry processes of SFTSV are grid protein-dependent endocytosis and endosome acidification [68].

The entry process of the virus first requires the recognition of cell surface receptors, among which Dendritic-cell-specific inter cellular adhesion molecule 3-grabbing non-integrin (DC-SIGN), heparan sulphate (HS), and Nonmuscle Myosin Heavy Chain IIA (NMMHCIIA) have been identified as involved in SFTSV entry [69]. Based on genome-wide CRISPR-Cas9 screening, Zhang et al. found that C-C motif chemokine receptor 2 (CCR2) enhances SFTSV binding by directly binding to SFTSV Gn, and knockout of CCR2 gene greatly reduces viral binding and infection, this suggests that CCR2 is the host entry receptor for SFTSV infection [70]. In the process of studying the entry mechanism of SFTSV, cytokines related to the internalization process have also been reported to be related to SFTSV entry, such as Sorting Nexin 11 (SNX11) and glucosylceramide synthase (UGCG). SNX11 is an intracellular transport protein mainly located in endosomal membrane. Liu et al. knocked out SNX11 gene by CRISPR-cas9 technology, and found that cells lacking SNX11 blocked the penetration of SFTSV from endolysosomes into the cytoplasm of host cells, indicating that SNX11 is an essential host cellular factor for SFTSV infection [71]. Drake et al. identified the host cell dependent glucose ceramide synthase for SFTSV entry through haploid forward genetic screening. Furthermore, inhibition of UGCG leads to the post-internalization phase of SFTSV entry being affected, with impaired transport and/or fusion of viral and host membranes leading to the accumulation of viral particles in enlarged cytoplasmic structures [72]. At present, although many attachment factors have been identified, the key receptors that determine cell orientation and entry are still unknown and need to be further studied.

### SFTSV infection in vivo

**Figure 4.**
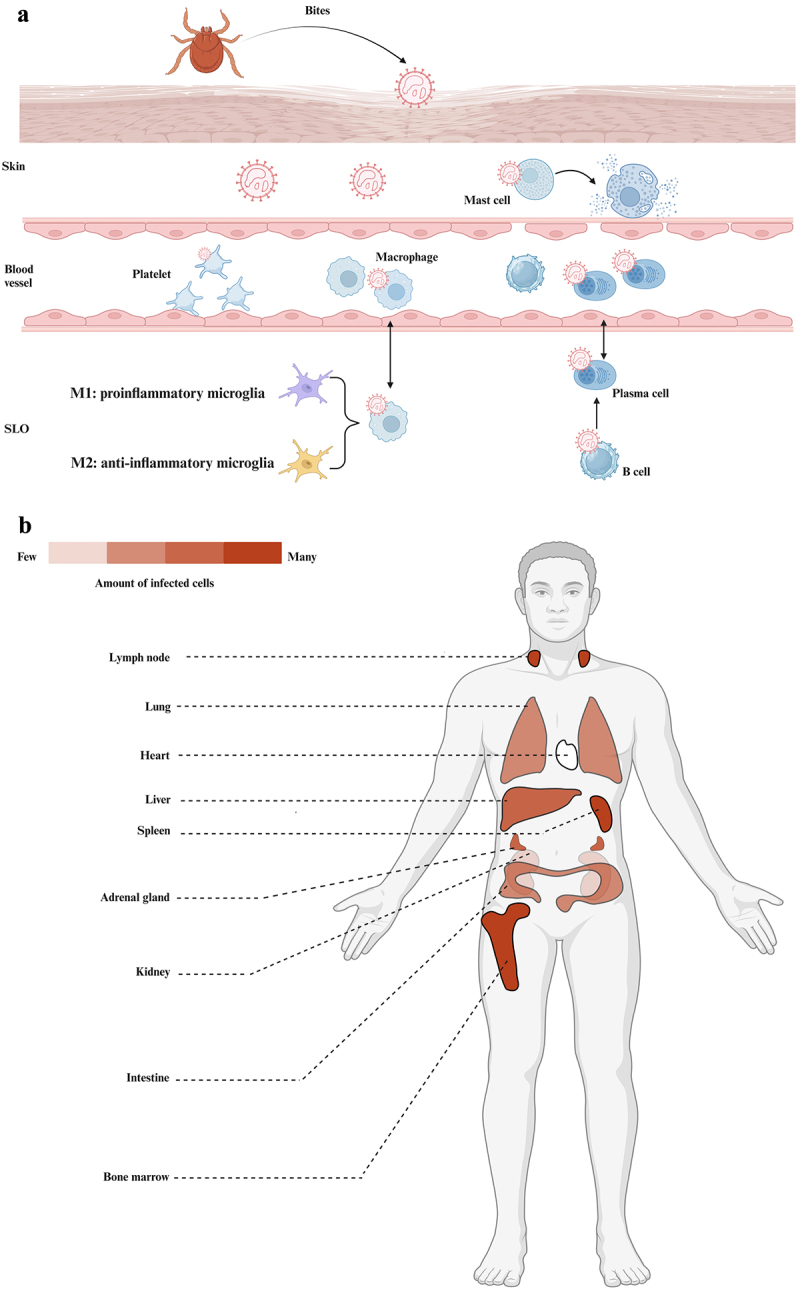
SFTSV infection and symptoms. (a), SFTSV is usually transmitted through the bite of a tick that carries the virus. Infection of skin resident cells, such as mast cell degranulation, resulted in damage to vascular endothelial cells. After the virus enters the bloodstream, platelets can carry and replicate SFTSV. SFTSV invades the secondary lymphoid organs closest to tick bite wounds, infecting cells such as B cells and macrophages, thereby achieving replication and transmission in the body (Created with BioRender.com). (b), Detection of infected cells in various organs. The number of cells infected in different organs varies. SFTSV-infected B-cell lineage lymphocytes are widely distributed in lymphatic and non-lymphoid organs, and occasionally these cells can be observed infiltrating the capillaries of the organ. The most infected cells were first detected in the bone marrow, spleen, lymph nodes, followed by the liver, adrenal glands, then the intestines, lungs, then the kidneys, and finally almost no infected cells were detected in the heart (created with BioRender.com).

Like other tick-borne viruses, SFTSV is transmitted to humans mainly by virus-carried tick bites. SFTSV-infected ticks attach to human skin and take blood after injecting SFTSV into the skin and blood, and the skin resident cells around the bitten site, such as immature Langerhans cells, epidermal dendritic cells (DCs), keratinocytes and mast cells (MCs) all may be the target of SFTSV infection [73]. Wang et al. found that SFTSV can cause MCs infection and degranulation, thereby releasing vasoactive mediators, chymase, and tryptase, which act directly on endothelial cells, disrupting their tight junctions and threatening the integrity of microvascular barrier, resulting in excessive microvascular permeability in human microvascular endothelial cells, causing bleeding and plasma leakage [74,75]. After the virus enters the blood, platelets are capable of harbouring and producing SFTSV particles. SFTSV bind platelet glycoprotein VI to potentiate platelet activation. In vitro mechanistic studies highlighted that the interaction between platelets with human THP-1 cells promoted the clearance of SFTSV and inhibited the production of cytokines in macrophages. However, unnecessary replication of SFTSV in macrophages, in turn, exacerbated the persistence of SFTSV circulation, thereby contributed to thrombocytopenia and other complications during SFTSV infection [76]. SFTSV replicates in various cell types in the body. Among these, infected monocytes avoid apoptosis and remain almost intact. Therefore, SFTSV within them can spread into the circulation via lymphatic drainage, causing viraemia [77,78]. In immunodeficient mouse models, immune cells in secondary lymphoid organs (SLOs) such as macrophages, immature B cells, and fibroblast reticular cells have been identified as targets for SFTSV infection [79]. The structure of the splenic white pulp and splenic follicles in the lymph nodes is mainly composed of B cells. Immature B cells are first affected by the interference of the spleen and lymph node viruses, producing cytokines that lead to widespread apoptosis and lymphocytopenia [80]. A post-mortem analysis of lymph nodes further confirmed that the majority of SFTSV-infected cells were B cells, specifically plasmablasts (PBs). PBs may first be infected by SFTSV in lymph nodes, then differentiate into plasma cells, and circulate in the blood. In contrast, SFTSV can independently infect plasma cells in lymph nodes and bloodstream [81]. The spleen is a major target organ for SFTSV, which can directly infect macrophages and remain latent in splenic macrophages [82]. Early in the course of SFTSV infection, macrophages may phenotypically differentiate to the M1 type (primarily promoting the inflammatory response). With prolonged infection, macrophages gradually tilt towards M2-type phenotypic differentiation (primarily inhibiting the inflammatory response), which promotes viral shedding and leads to viral dissemination [83]. While SFTSV can hijack macrophages for replication, macrophages can also inhibit the growth of the virus and eventually clear it in the body [82]. This suggests that SFTSV can be cleared in immunocompetent individuals, but immunosuppressed patients may suffer multiple organ dysfunction or death as the virus effectively proliferates [4] (Figure 4).

### Features of clinical infection

The clinical features of SFTSV infection are characterized by sudden onset of fever and respiratory or gastrointestinal symptoms, followed by a gradual decline in platelets and leukocytes. The typical infectious process has four distinct periods: incubation, fever, multiple organ failure, and convalescence [4]. Diarrhoea, dyspnoea, haemorrhagic signs, and neurological symptoms are important predictors for mortality in SFTS cases, with neurological symptoms being the most critical. However, most neurological symptoms often appear in late course of disease, therefore the other three manifestations (diarrhoea, dyspnoea and haemorrhagic signs) can serve as additional predictors of death [84].

The incubation period is approximately 1–2 weeks prior to onset of illness, and there is usually a history of tick bites or/and close contact with patients with confirmed SFTS. The length of the incubation period is related to viral load, exposure, and personal susceptibility [85]. The incubation period is generally considered to be 5–14 days for vector tick transmission and 7–12 days for blood-to-person transmission.

The febrile period lasts for 5–7 days and is characterized by flu-like symptoms, such as acute onset and fever, with a temperature of up to 38°C or above, severe patients may experience sustained high fever, up to 40°C or above [86,87], simultaneously accompanied by headache, fatigue, muscle pain, and gastrointestinal symptoms (e.g. loss of appetite, nausea, vomiting diarrhoea). Marked thrombocytopenia and leucopenia, and lymph node enlargement usually occur during this period as well [88]. At this stage, the SFTSV virus is most active and can be an important criterion for clinical diagnosis.

The multi-organ failure period mostly occurs about 5 days after the onset and lasts for 7–14 days. This period progresses rapidly, with rapid damage to multiple organs of the body, which can involve the liver, lungs, and kidneys (Figure 4b), and mostly overlaps with the febrile period [89]. Some patients experience central nervous system symptoms, manifested as impaired consciousness (apathy, lethargy, coma), muscle tremors, convulsions, and irritability. In addition, acute encephalopathy or encephalitis is also a common complication of the central nervous system in patients with SFTS [90,91]. Hemorrhagic tendencies are also more common at this stage, manifested by skin petechiae, gingival bleeding, gastrointestinal bleeding, and pulmonary haemorrhage, which may be related to thrombocytopenia and anticoagulant depletion [89,92]. It was found that the vast majority of patients with SFTS in the mortality group had puncture-site bruising, diffuse intravascular coagulation (DIC), gastrointestinal or respiratory bleeding, while the incidence of these symptoms was very low in the survival group of patients [93]. Serum viral loads declined progressively in most patients during this period, but remained high in deceased patients. Concentrations of other important biomarkers such as alanine aminotransferase (ALT), aspartate aminotransferase (AST), lactate dehydrogenase (LDH), creatine kinase (CK), and creatine kinase MB fraction (CK-MB) also showed significant differences between survivors and deceased cases [94,95]. This stage is particularly important for the recovery of SFTS patients and is a critical stage of treatment.

The majority (85%) of patients have a favourable prognosis and enter the convalescence phase 11–19 days after onset of illness. The patient’s clinical symptoms and various laboratory test indicators gradually returned to normal, and the serum viral load continued to decrease [89,94]. However, elderly patients with SFTS generally have poor physical resistance and are often accompanied by other clinical complications, which may result in slower improvement or even exacerbation of the disease and prolonged recovery time [95].

Among the clinical complications, the incidence of pulmonary infections was found to be 100% in the critically ill group of patients and fungal infections can also be associated with it [96]. Acute encephalopathy/encephalitis as the central nervous system manifestation of SFTS is a common complication [82]. Necrotizing lymphadenitis of systemic lymphatic tissues was revealed as a rare complication of SFTS in the study of HIRAKI et al [97]. SFTS can also lead to myocardial dysfunction as well. The incidence of necrotizing lymphadenitis of systemic lymphatic tissues is a rare complication of SFTS [98].

## Diagnosis and prevention

### Diagnosis

According to the criteria of the Guidelines for Prevention and Control of Fever with Thrombocytopenia Syndrome (2010 edition) issued by the former Ministry of Health, China. Confirmation of the diagnosis of clinically suspected cases of SFTS needs to be combined with the patient’s epidemiologic history (cases have a history of hills during the epidemic season, (history of living, working or traveling in forested or mountainous areas during the epidemic season, or history of bee bite two weeks before the onset of the disease), fever (>37.5°C) and other symptoms. 37.5°C), clinical manifestations such as fever (>37.5°C), and obvious peripheral blood thrombocytopenia (<100.0 × 10^9^/L) and leukopenia (<4.0 × 10^9^/L), and other laboratory tests [99]. Currently, laboratory tests are mainly used for virus isolation, viral nucleic acid testing and serum antibody testing (Table 1). Virus isolation is commonly used for laboratory operations and is not suitable for rapid clinical diagnosis due to its high technical requirements and time-consuming nature. Reverse transcriptase polymerase chain reaction (RT-PCR) is a highly specific, sensitive, and rapid laboratory diagnostic method for SFTSV infection, and is currently a commonly used method for early detection of pathogens [100]. SFTSV nucleic acid cannot yet be carried out in some areas due to limitations in laboratory technology, personnel or laboratory environment. Zhou et al. developed a rapid isothermal real-time reverse transcription recombinase polymerase amplification (RT-RPA) assay for SFTSV, which can quickly produce results within 15 minutes at 39°C, and the RT-RPA assay can be used for the field detection of SFTSV in resource-constrained settings [101]. Yoshikawa et al. developed a sensitive and specific conventional one-step RT-PCR method and a quantitative one-step RT-PCR to detect both strains to overcome the above issues [102]. Huang et al. developed a reverse transcription loop-mediated isothermal amplification (RT-LAMP) technique to rapidly identify the new bunyavirus with 99% sensitivity and 100% specificity [103]. Baek et al. also demonstrated that RT-LAMP could provide a rapid diagnosis within 30–60 minutes, with sensitivity 10 times higher than conventional RT-PCR [104]. Zhang et al. combined CRISPR-Cas13a nucleic acid detection with RAA to establish a simple, rapid, highly sensitive, and highly specific nucleic acid test for SFTSV, which provides a new method for on-site diagnosis [105]. Neutralization testing is the gold standard for viral diagnosis, but it is laborious and expensive and requires manipulation of live viruses, which can only be performed in biosafety laboratories.

**Table 1.** Diagnostic tests for detection and control of DBV infection.

Test	Detection target	Interpretation	Advantages	Limitations	Reference
Virus isolation culture	Live virus	Isolate the virus from cell cultures	Simple; highly specific; can be quantitative	The virus has little or no effect on cytopathies and needs to be confirmed by electron microscopy or serology	Liu et al. [4]
Viral nucleic acid testing	Viral RNA	Include RT-PCR,RT-RPA, RT-LAMP, CRISPR-Cas13a/RAA, et al.	Highly sensitive and specific diagnostic tools	Complex methods are required to detect amplified products and sophisticated instruments	Yoshikawa et al.[100], Huang et al. [102], Baek et al. [103], Zhang et al.[104]
Serum antibody testing	IgM/IgG antibody	Include Neutralization testing,IFA ELISA, ICA, et al.	It can be used as an adjunct to diagnosis when the detection of viral nucleic acid is negative, Retrospective testing for DBV infection	Skilled technicians, subjective interpretation, and time-consuming	Liu et al. [4], Ningning et al. [105]

RT-PCR: Reverse Transcription-Polymerase Chain Reaction, RT-RPA: Reverse Transcription Recombinase Polymerase Amplification, RT-LAMP: Reverse Transcription Loop-Mediated Isothermal Amplification, CRISPR-Cas13a: Clustered Regularly Interspaced Short Palindromic Repeats associated protein 13a, CRISPR-RAA: Clustered Regularly Interspaced Short Palindromic Repeats-Recombinase-Aided Amplification, IFA: Immunofluorescence Analysis, ELISA: Enzyme-Linked Immunosorbent Assay, ICA: Immunochromatography assay.

Immunofluorescence assay (IFA) or enzyme-linked immunosorbent assay (ELISA) are effective diagnostic methods for detecting virus-specific IgM and IgG in serum after 7 days of onset [4]. IFA requires a large amount of virus, skilled technicians, involves subjective interpretation, and is also time-consuming [106]. The ELISA technique is less costly and time-consuming, and a recombinant nucleoprotein-based double antigen sandwich ELISA has been developed for the detection of SFTSV antibodies in humans and animals [107]. Immunochromatographic assays (ICA) are suitable for rapid, in situ, inexpensive SFTSV detection, and commercially available reagents also have good sensitivity and specificity for the diagnosis of specific SFTSV IgG and IgM antibodies [108].

### Differential diagnosis

SFTS should be differentiated from human granulocytic anaplasmosis (HGA), haemorrhagic fever with renal syndrome (HFRS), dengue fever, Lyme Disease (LD), Rickettsiosis, Crimean-Congo Haemorrhagic Fever (CCHF), Babesiosis, Malaria and primary immunologic thrombocytopenic purpura (ITP).

HGA, a tick-borne infection caused by *Anaplasma phagocytophilum*. Patients with HGA have clinical symptoms and epidemiologic history similar to those of SFTS [108]. However, proteinuria, haematuria, and tubular urine can be seen in routine urinalysis of patients with HGA, and the diagnosis can be confirmed by serologic and pathogenic tests.

HFRS is a zoonotic disease transmitted by several rodent species. Similar to SFTS, HFRS patients also exhibit fever and gastrointestinal symptoms, but they have symptoms caused by systemic capillary damage, characterized by typical three pains (headache, back pain, orbital pain) and three redness (redness on the face, neck, and upper chest). In addition, the patient’s kidney damage is prominent, which was manifested as hypotensive shock and oliguria (less than 400 ml of urine in 24 hours) [109]. In addition, the patient’s urine routine examination showed an increasing trend of white blood cells, contrary to SFTS.

Dengue fever is an acute infectious disease caused by mosquito borne transmission of the dengue virus. The symptoms of fever and vascular involvement in dengue fever patients are similar to those of SFTS, but the patient’s fever type is bimodal or saddle shaped fever, and exhibit severe muscle and bone joint soreness [110]. Diagnosis can be made based on the epidemiological characteristics of epidemic area and rainy season, combined with serologic and pathogenic tests.

ITP is an acquired autoimmune disease. It is the most common haemorrhagic disease that causes a decrease in platelet count [104]. The patient’s bone marrow examination shows an increase or normal number of megakaryocytes, with maturation disorders.Lyme disease is a zoonotic disease transmitted by ticks. Similar to SFTS, Lyme disease patients also present with fever, headache, and muscle pain. However, Lyme disease is characterized by a distinctive erythema migrans rash that expands over time. Neurological symptoms such as facial palsy and meningitis are also common in Lyme disease but are rare in SFTS [111].

Rickettsiosis is caused by bacteria of the Rickettsia genus and is transmitted through arthropods like ticks, fleas, and lice. Similar to SFTS, rickettsiosis patients also present with fever and headache. However, they also develop a characteristic rash that begins on the wrists and ankles and spreads to the trunk, which is uncommon in SFTS. Patients may also experience muscle pain and chills. Diagnosis can be confirmed by serological or PCR testing [112].

CCHF is a viral haemorrhagic fever caused by the Crimean-Congo Haemorrhagic Fever virus, transmitted through ticks. CCHF patients exhibit clinical symptoms similar to SFTS, such as fever, headache, muscle pain, dizziness, and gastrointestinal symptoms. However, CCHF patients typically present with more severe haemorrhagic manifestations, including petechiae, ecchymoses, and mucosal bleeding, which are uncommon in SFTS patients. Additionally, CCHF can be distinguished from SFTS by laboratory detection of CCHF virus RNA in the patient’s blood [113].

Babesiosis is caused by the Babesia parasite and transmitted through ticks. Similar to SFTS, babesiosis patients present with fever and fatigue. However, babesiosis also manifests with haemolytic anaemia, jaundice, and splenomegaly, which are not common in SFTS. Diagnosis can be confirmed through blood smear examination or PCR [114].

Malaria is caused by Plasmodium parasites and transmitted by Anopheles mosquitoes. Similar to SFTS, malaria patients present with high fever and chills. However, malaria has a characteristic periodic fever pattern and can lead to haemolytic anaemia and splenomegaly. The gold standard for malaria diagnosis is the identification of Plasmodium parasites in a blood smear using microscopic examination. Rapid diagnostic tests (RDTs) are also widely used for quick and effective malaria diagnosis [115,116].

Leptospirosis is a zoonotic disease caused by Leptospira bacteria and transmitted through contact with contaminated water or soil. Similar to SFTS, leptospirosis patients present with fever and headache. However, leptospirosis is also characterized by muscle pain (particularly in the calves), jaundice, and kidney failure. Routine urinalysis shows an increase in white blood cells, which is contrary to SFTS [117].

### Prevention

#### Vaccine development

There is no vaccine or chemoprophylaxis for SFTSV. Development of a vaccine is one of the most effective ways to target SFTSV infection. Vaccines should be highly effective in preventing infection, morbidity, or severe disease. Several SFTSV vaccine candidates have been developed and have demonstrated effectiveness in animal models (Table 2). Vaccine candidates include live attenuated vaccines, recombinant viral vector vaccines, protein subunit vaccines, and DNA vaccines.

**Table 2.** DBV vaccine candidates.

Vaccine Type	DBV lineage	DBV strain	Animal models	Advantages	Limitations	Status	Reference
Live attenuated virus vaccine	genotype D	Hubei, China, 2010	ferrets	Genes are stable, and can induce complete protection against lethal DBV challenge	Risk of re-intensification of virulence	Preclinical development stage	Yu et al. [118]
Viral vector vaccines	genotype D	Anhui, China, 2010	mice	Protective neutralizing antibody that causes high titres, providing complete protection against lethal attack by DBV	The potential role of cellular immunity against viral antigens in complete protection has not been studied	Preclinical development stage	Dong et al. [119]
DNA Vaccines	genotype D	China, 2011	mice	Relatively easy to develop, capable of inducing broad immunity to multiple antigens, stimulating T cell and antibody immunity	Low immunogenicity and require large amounts of DNA or other excipients to produce a robust immune response in humans	Preclinical development stage	Gary and Weiner [120], Kang et al. [121]
Inactivated vaccines	Genotype A	China, 2011	mice	conferred efficacious protective immune responses from DBV	Short immunization timeframe: antibodies produced weaken over time, requiring multiple vaccinations	Preclinical development stage	Sabbaghi et al.[122], Aqian [123]

##### Live attenuated virus vaccine

Live attenuated vaccines consist of live viruses with low pathogenicity. Yu et al. investigated the effectiveness of attenuated mutant viruses as anti-SFTSV vaccine candidates [118]. None of the ferrets infected with attenuated mutant viruses (rHB2912aaNSs) died, whereas ferrets infected with genotype D (the same genotype as rHB29) isolated from South Korea lost more than 10% of their body weight, had an elevated body temperature, and had a 100% mortality rate. After 58 days of infection with rHB2912aaNSs, ferrets had neutralization titres exceeding 2.5 FRNT_50_ (10log_2_) and gained complete protection from SFTSV challenge. Given their diminished pathogenicity and ability to mimic natural infection, live attenuated vaccines hold promise as a countermeasure against SFTSV.

##### Viral vector vaccines

Viral vectors carry genes that express desired antigens to prevent infectious viruses. Many viral vectors serve as potential drug candidates for the prevention of SFTSV infections. Dong et al. developed a recombinant vesicular stomatitis virus (rVSV) expressing SFTSV Gn/Gc, called rVSV-SFTSV/AH12-GP, as a viral vector vaccine [119]. Compared to the original rVSV-G (rVSV), rVSV-SFTSV/AH12-GP has reduced pathogenicity in IFNAR-/- mice. Sera from immunized mice neutralized SFTSV at a titre of 4 ~ 8, and protection was obtained against 1000 LD_50_ SFTSV challenge, suggesting that the rVSV platform vaccine is a promising candidate for the development of SFTSV vaccines.

##### DNA vaccines

DNA vaccines are easy to design, stable, inexpensive to produce, and effective in preventing a wide range of viral infectious diseases, such as those caused by HIV-1, Zika virus, and Ebola virus [120]. Kang et al [121]. integrated the Gn, Gc, NP, and NSs genes into a single vector (pSFTSV), and added the IL-12 gene (pSFTSV IL-12) to enhance cell-mediated immunity. The pSFTSV IL-12 group exhibited Gn- and NP-specific CD4^+^ and CD8^+^ T cell responses compared to the vector group. The transfected pSFTSV-IL-12 mice had 100% protection from SFTSV challenge. This study demonstrates that DNA vaccines can protect IFNAR-/- mice from SFTSVinfection to some extent.

##### Inactivated vaccines

Inactivated vaccines are safe and effective in preventing disease [122]. These vaccines are made by propagating the pathogen in a culture medium and then inactivating the pathogen using chemicals such as ß-propiolactone, formaldehyde, or detergents [123]. Li et al. [123] studied ß-propiolactone inactivation of SFTSVviruses in experiments in which BALB/c and C57BL/6 mice were inoculated, and all the vaccinated mice developed, two weeks after the last inoculation, SFTSV-specific IgG and neutralizing antibodies. In addition, no virus was detected in the sera of mice vaccinated with either medium- or high-dose vaccines after SFTSVchallenge, suggesting that inactivated vaccines are ideal candidates for the prevention of SFTSV infection.

### Vector prevention

Ticks are the main vector of SFTSV, and SFTS outbreaks can be controlled using traditional tick control methods. Tick control is mainly based on the application of chemical insecticides. For example, chemical repellents achieve repellency by volatilizing some special odour that protects humans and animals from tick bites for a certain period of time [124]. The use of acaricides kills ticks in the environment in the autochthonous stage or parasitic on the host. In the western United States, certain plant sprays are used to control ticks in order to reduce attacks on humans. Unfortunately, long-term large-scale use of chemicals not only leads to increased tick resistance, but also causes severe environmental contamination and potential harm to human health. This approach has not been widely promoted due to environmental contamination and the high cost of large-scale treatment [125]. Intratick pathogen surveillance is also one of the most important tools for the prevention and control of tick-borne diseases [126]. To realize the effectiveness of tick control strategies, the dynamic associations between disease pathogens, vertebrate hosts, vector ticks and the environment should be systematically understood. Countries that have eradicated tick-borne diseases should prevent the invasion of exotic ticks by implementing relatively stringent quarantine measures during the actual livestock breeding process, effectively identify the presence of tick invasion in uninfected feeding areas by taking into account the climatic, geographic and ecological characteristics of the feeding areas, and prepare in advance for the threat of tick-borne diseases once they are detected. In addition, public health education should be actively pursued, such as popularizing knowledge of SFTS in areas where outbreaks occur or where they are endemic; Emphasis should be placed on the safe and proper handling of animals, as well as monitoring and reporting; As SFTS is an emerging disease with limited reporting, there is a need to strengthen the SFTS epidemiological surveillance and early warning system.

## Management

There is no specific treatment for this disease, mainly symptomatic supportive treatment. Patients should rest in bed, consume liquid or semi liquid food, and drink plenty of water. Close monitoring of vital signs and urine output. Patients who cannot eat or are in serious condition should supplement calories in time to ensure the of water, electrolyte, and acid-base, especially for those with hyponatraemia [104]. Individuals with high fever should physically cool down and, if necessary, use medication to reduce fever. For patients with significant bleeding or significant decrease in platelets (such as < 30 × 10^9^/L), plasma or platelets can be transfused. In patients with severe neutropenia (<1 × 10^9^/L), it is recommended to use granulocyte colony-stimulating factor. Secondary bacterial and fungal infections should be treated with sensitive antibiotics. Attention should also be paid to the treatment of the underlying disease. At present, there is no evidence to prove the therapeutic effect of glucocorticoids, and they should be used with caution.

At present, potential therapeutic drugs against SFTSVmainly includes:

Ribavirin is a nucleotide analog with inhibitory activity against a variety of DNA and RNA viruses [127]. According to a large-scale epidemiologic study conducted in China (including 2,096 patients with laboratory-confirmed SFTS between 2011–2017), ribavirin therapy reduced case fatality rate (CFR) from 6.25% to 1.16% in patients with viral loads < 1 × 10^6^ copies/mL. However, no significant treatment effect was observed in patients with viral loads > 1 × 10^6^ copies/mL [94]. This finding suggests that the therapeutic effect of ribavirin on SFTSV infection is dependent on viral load. Currently, the efficacy of ribavirin in treating patients with SFTS in the clinic is still under further investigation [128,129].

Favipiravir (*T*-705), an RNA-dependent RNA polymerase inhibitor, has been approved for the treatment of novel and recurrent influenza in Japan, and is currently undergoing Phase 3 clinical trials in the United States [130]. *T*-705 has been reported to exhibit higher anti- SFTSV efficacy than ribavirin in vitro and animal models [131]. Li et al. randomized 145 patients with laboratory-confirmed SFTS into a control group (supportive care) and a treatment group (oral *T*-705 combined with supportive care), to assess the efficacy and safety of *T*-705 in the treatment of SFTS (Chinese Clinical Trial Registry website, number ChiCTR1900023350). The results showed that *T*-705-treated group had a shorter time to viral clearance, lower incidence of haemorrhagic signs, and faster recovery from laboratory abnormalities than the control group. And no significant serious adverse events were observed in patients during *T*-705 treatment. More importantly, in the low-baseline viral load subgroup (RT-PCR cycle-threshold ≥26), *T*-705 treatment significantly reduced CFR from 11.5 to 1.6% (p = 0.029). No between-group differences was observed in patients with high-baseline viral loads, with moderately reduced in the *T*-705-treated group, perhaps due to the small sample size and relatively low drug dose. In vitro and animal experiments showed that the antiviral effect of *T*-705 was induced proportionally by the SFTSV mutation rates. Mutation analysis of serum samples from patients in the *T*-705 treatment group further supported the antiviral effect of *T*-705. The results of this study support the idea that favipiravir may be an effective drug for treating SFTS patients [132].

Calcium channel blockers (CCBs) can reduce intracellular Ca^2+^ levels and are widely used in the treatment of various cardiovascular diseases, including hypertension, angina, and supraventricular arrhythmias. Recent studies have shown that CCBs exhibits antiviral activity against Ebola [133], Marburg [134], and West Nile viruses [135]. Li et al. screened an FDA-approved drug library that contained 700 drug compounds and identified benidipine hydrochloride, a calcium channel blocker (CCB), inhibited SFTSV replication in vitro. Further experiments have shown that a large number of CCBs, including nifedipine, can inhibit SFTSV infection. The anti-SFTSV efficacy of two CCBs was further confirmed in C57BL/6 and humanized mouse models, and CCB treatment was found to reduce viral load and mortality. Importantly, in a retrospective clinical study of 2087 SFTS patients, nifedipine was found to enhance viral clearance, improve clinical recovery, and significantly reduce the mortality rate of SFTS patients by 5-fold. Due to the limited use of benidipine hydrochloride in clinical practice in China, there is currently no clinical data available to evaluate its efficacy in treating SFTS patients. These studies suggest that CCB may be an effective countermeasure for the treatment of SFTSV infection [136].

Some finished drugs commonly used to treat other diseases can also inhibit SFTSV in vivo or in vitro, such as the proteasome inhibitor bortezomib (PS-341) blocked SFTSV infection by affecting virus infectivity,replication, and release [137]. Antimalarial agent amodiaquine derivative C-90 can effectively inhibit viral replication in a dose-dependent manner, and its 50% effective concentration (EC_50_) value is comparable to or slightly better than that of favipiravir [138]. The antifungal drug anidulafungin inhibits the SFTSV entry process and affects the stability of viral particles under high-dose conditions in vitro. In addition anidulafungin improved the outcome of SFTSV infection and reduced lethality in mice [139]. Some natural chemical products are also useful in the treatment of SFTS. Motohiko et al. evaluated the effect of caffeic acid (CA, a coffee-related organic acid with antiviral properties) on SFTSV infection, and found that CA inhibited viral infection and transmission primarily by inhibiting the binding of SFTSV to cells [140]. In short, the above drugs have certain interference effects on SFTSV replication and transmission, and are expected to be therapeutic or prophylactic drugs for SFTS, or provide some help in the development of specific therapeutic drugs.

## Outlook

Over time, more strains of SFTSV may evolve. What is certain is that SFTS cases are increasing year by year (especially in East Asia) and the region of the epidemic is still expanding. In the decade since the discovery of SFTSV in China, a great deal of research has been conducted on the viral vectors, the ways in which the virus invades organisms, and the drugs that target the virus [2,12,94]. However, there are still many issues to be explored, such as the pathogenesis of SFTSV and the development of efficient vaccines and specific drugs [141]. SFTSV, as a segmented RNA virus, has a genome prone to mutation and recombination. In addition, climatic factors such as barometric pressure, temperature and relative humidity, and complex ecological factors such as topography, geomorphology, grasslands or forests also affect vector and host activities and distribution, which in turn promote the continuous adaptive evolution of SFTSV [34,36,37]. Global warming has obvious ecological advantages for the expanding spread of SFTSV, which poses a serious threat to global public health. There is an urgent need to better predict the dynamic patterns of SFTSV, vector and host cycles by means of advanced remote sensing technologies, global positioning systems, geographic information systems and spatial epidemiology.

SFTSV detection methods are diverse and applicable in different scenarios. The qRT-PCR methods have been widely used in clinics and CDCs [92,142]. ELISAs and IFAs are more sensitive than the neutralization assay for detecting SFTSV -specific IgG/M antibodies. The ICA is suitable for rapid detection in the field. The SFTSV neutralization assay is suitable for detecting neutralizing antibodies in the sera of convalescing patients. Therefore. Selection of appropriate assays is conducive to efficient SFTSV diagnosis. There is no commercial vaccine for SFTSV, and vaccine development faces many challenges [143]. For example, a universal vaccine that induces cross-protective immunity to different SFTSV genotypes. The role of climatic factors, cross-species transmission, and the development of vaccines and new drugs may be hotspots for future research.

With many academics and clinicians actively sharing their experiences, researchers are also committed to developing effective therapeutic regimens. SFTSV is recognized by the virological and medical communities as an emerging and resurgent pathogen, but has not yet received sufficient attention from governments and relevant international organizations. Research on SFTSV and other viruses transmitted by arthropods often needs to be urgently prioritized and upgraded if we want to prepare for future pandemics and, ideally, prevent them from occurring. We should enhance our understanding of SFTSV and improve clinical management, as well as infection prevention and control skills, especially among public health workers. Finally, we should initiate global cooperation for clinical research to test the efficacy and safety of SFTSV vaccines and antiviral drugs.

## Data Availability

Data availability is not applicable to this article as no new data were created or analysed in this study.

## References

[1] Casel MA, Park SJ, Choi YK. Severe fever with thrombocytopenia syndrome virus: emerging novel phlebovirus and their control strategy. Exp Mol Med. 2021;53(5):713–19. doi: 10.1038/s12276-021-00610-1 33953322 PMC8178303

[2] Yu XJ, Liang MF, Zhang SY, et al. Fever with thrombocytopenia associated with a novel bunyavirus in China. N Engl J Med. 2011;364(16):1523–1532. doi: 10.1056/NEJMoa1010095 21410387 PMC3113718

[3] Li YH, Huang WW, He WQ, et al. Longitudinal analysis of immunocyte responses and inflammatory cytokine profiles in SFTSV-infected rhesus macaques. Front Immunol. 2023;14:1143796. doi: 10.3389/fimmu.2023.1143796 37033979 PMC10073517

[4] Liu Q, He B, Huang SY, et al. Severe fever with thrombocytopenia syndrome, an emerging tick-borne zoonosis. Lancet Infect Dis. 2014;14(8):763–772. doi: 10.1016/S1473-3099(14)70718-2 24837566

[5] Sun Z, Cheng J, Bai Y, et al. Architecture of severe fever with thrombocytopenia syndrome virus. Protein & Cell. 2023;14(12):914–918. doi: 10.1093/procel/pwad019 37038326 PMC10691843

[6] Lei XY, Liu MM, Yu XJ. Severe fever with thrombocytopenia syndrome and its pathogen SFTSV. Microbes Infect. 2015;17(2):149–154. doi: 10.1016/j.micinf.2014.12.002 25498868

[7] Hornak KE, Lanchy JM, Lodmell JS. RNA encapsidation and packaging in the phleboviruses. Viruses. 2016;8(7):194. doi: 10.3390/v8070194 27428993 PMC4974529

[8] Sharma D, Kamthania M. A new emerging pandemic of severe fever with thrombocytopenia syndrome (SFTS). Virusdisease. 2021;32(2):220–227. doi: 10.1007/s13337-021-00656-9 33942022 PMC8082055

[9] Wang P, Liu L, Liu A, et al. Structure of severe fever with thrombocytopenia syndrome virus L protein elucidates the mechanisms of viral transcription initiation. Nat Microbiol. 2020;5(6):864–871. doi: 10.1038/s41564-020-0712-2 32341479

[10] Tran XC, Yun Y, Van an L, et al. Endemic severe fever with thrombocytopenia syndrome, Vietnam. Emerg Infect Dis. 2019;25(5):1029–1031. doi: 10.3201/eid2505.181463 31002059 PMC6478219

[11] Lin TL, Ou SC, Maeda K, et al. The first discovery of severe fever with thrombocytopenia syndrome virus in Taiwan. Emerging Microbes & Infect. 2020;9:148–151. doi: 10.1080/22221751.2019.1710436 PMC6968498 31918622

[12] Zhang Y, Huang Y, Xu Y. Antiviral treatment options for severe fever with thrombocytopenia syndrome infections. Infect Dis Ther. 2022;11(5):1805–1819. doi: 10.1007/s40121-022-00693-x 36136218 PMC9510271

[13] Kwak JE, Kim YI, Park SJ, et al. Development of a SFTSV DNA vaccine that confers complete protection against lethal infection in ferrets. Nat Commun. 2019;10(1):3836. doi: 10.1038/s41467-019-11815-4 31444366 PMC6707330

[14] Kim D, Lai CJ, Cha I, et al. Current progress of severe fever with thrombocytopenia syndrome virus (SFTSV) vaccine development. Viruses. 2024;16(1):128. doi: 10.3390/v16010128 38257828 PMC10818334

[15] Chen QL, Zhu MT, Chen N, et al. [Epidemiological characteristics of severe fever with thtrombocytopenia syndrome in China, 2011–2021]. Zhonghua Liu Xing Bing Xue Za Zhi. 2022;43(6):852–859. doi: 10.3760/cma.j.cn112338-20220325-00228 35725341

[16] Rattanakomol P, Khongwichit S, Linsuwanon P, et al. Severe fever with thrombocytopenia syndrome virus infection, Thailand, 2019–2020. Emerg Infect Dis. 2022;28(12):2572–2574. doi: 10.3201/eid2812.221183 36418010 PMC9707585

[17] Zohaib A, Zhang J, Saqib M, et al. Serologic evidence of severe fever with thrombocytopenia syndrome virus and related viruses in Pakistan. Emerg Infect Dis. 2020;26(7):1513–1516. doi: 10.3201/eid2607.190611 32568060 PMC7323538

[18] Wu Y, Gao GF. Severe fever with thrombocytopenia syndrome virus expands its borders. Emerging Microbes & Infect. 2013;2:e36. doi: 10.1038/emi.2013.36 PMC3697302 26038472

[19] Hu YY, Zhuang L, Liu K, et al. Role of three tick species in the maintenance and transmission of severe fever with thrombocytopenia syndrome virus. PLOS Negl Trop Dis. 2020;14(6):e0008368. doi: 10.1371/journal.pntd.0008368 32520966 PMC7307786

[20] Seo JW, Kim D, Yun N, et al. Clinical update of severe fever with thrombocytopenia syndrome. Viruses. 2021;13(7):1213. doi: 10.3390/v13071213 34201811 PMC8310018

[21] Zhang X, Zhao C, Cheng C, et al. Rapid spread of severe fever with thrombocytopenia syndrome virus by Parthenogenetic Asian Longhorned Ticks. Emerg Infect Dis. 2022;28(2):363–372. doi: 10.3201/eid2802.211532 35075994 PMC8798674

[22] Wang S, Li J, Niu G, et al. SFTS virus in ticks in an endemic area of China. Am J Trop Med Hyg. 2015;92(4):684–689. doi: 10.4269/ajtmh.14-0008 25711611 PMC4385759

[23] Yabsley MJ, Thompson AT. Haemaphysalis longicornis (Asian longhorned tick). Trends Parasitol. 2023;39(4):305–306. doi: 10.1016/j.pt.2022.12.007 36631384

[24] Luo LM, Zhao L, Wen HL, et al. Haemaphysalis longicornis ticks as reservoir and vector of severe fever with thrombocytopenia syndrome virus in China. Emerg Infect Dis. 2015;21(10):1770–1776. doi: 10.3201/eid2110.150126 26402039 PMC4593435

[25] Yun Y, Heo ST, Kim G, et al. Phylogenetic analysis of severe fever with thrombocytopenia syndrome virus in South Korea and migratory bird routes between China, South Korea, and Japan. Am J Trop Med Hyg. 2015;93(3):468–474. doi: 10.4269/ajtmh.15-0047 26033016 PMC4559681

[26] Chen C, Li P, Li KF, et al. Animals as amplification hosts in the spread of severe fever with thrombocytopenia syndrome virus: a systematic review and meta-analysis. Int J Infect Dis. 2019;79:77–84. doi: 10.1016/j.ijid.2018.11.017 30500443

[27] Huang XY, Du YH, Wang HF, et al. Prevalence of severe fever with thrombocytopenia syndrome virus in animals in Henan Province, China. Infect Dis Poverty. 2019;8(1):56. doi: 10.1186/s40249-019-0569-x 31230595 PMC6589873

[28] Kirino Y, Yamamoto S, Nomachi T, et al. Serological and molecular survey of tick-borne zoonotic pathogens including severe fever with thrombocytopenia syndrome virus in wild boars in Miyazaki Prefecture, Japan. Vet Med Sci. 2022;8(2):877–885. doi: 10.1002/vms3.696 34953052 PMC8959263

[29] Zhao C, Zhang X, Si X, et al. Hedgehogs as amplifying hosts of severe fever with thrombocytopenia syndrome virus, China. Emerg Infect Dis. 2022;28(12):2491–2499. doi: 10.3201/eid2812.220668 36417938 PMC9707592

[30] Rainey T, Occi JL, Robbins RG, et al. Discovery of haemaphysalis longicornis (Ixodida: Ixodidae) parasitizing a sheep in new Jersey, United States. J Med Entomol. 2018;55(3):757–759. doi: 10.1093/jme/tjy006 29471482

[31] Wormser GP, McKenna D, Piedmonte N, et al. First recognized human bite in the United States by the Asian longhorned tick, haemaphysalis longicornis. Clin Infect Dis. 2020;70(2):314–316. doi: 10.1093/cid/ciz449 31150055

[32] Dong M. Dynamic distribution and transmission risk prediction of severe fever with thrombocytopenia syndrome [chinese master’s thesis]. Academy of Military Sciences; 2020. Available from: https://www.cnki.net/

[33] Ji SR, Byun HR, Rieu MS, et al. First detection of Bandavirus dabieense in ticks collected from migratory birds in the Republic of Korea. Acta Trop. 2024;257:107279. doi: 10.1016/j.actatropica.2024.107279 38871069

[34] Barrett B, Charles JW, Temte JL. Climate change, human health, and epidemiological transition. Prev Med. 2015;70:69–75. doi: 10.1016/j.ypmed.2014.11.013 25434735 PMC4342988

[35] Heath A. Biology, ecology and distribution of the tick, haemaphysalis longicornis Neumann (Acari: Ixodidae) in New Zealand. N Z Vet J. 2016;64(1):10–20. doi: 10.1080/00480169.2015.1035769 25849758

[36] Gilbert L. The impacts of climate change on ticks and tick-borne disease risk. Annu Rev Entomol. 2021;66(1):373–388. doi: 10.1146/annurev-ento-052720-094533 33417823

[37] Ogden NH, Lindsay LR. Effects of climate and climate change on vectors and vector-borne diseases: ticks are different. Trends Parasitol. 2016;32(8):646–656. doi: 10.1016/j.pt.2016.04.015 27260548

[38] Miao D, Liu MJ, Wang YX, et al. Epidemiology and ecology of severe fever with thrombocytopenia syndrome in China, 2010‒2018. Clin Infect Dis. 2021;73(11):e3851–e3858. doi: 10.1093/cid/ciaa1561 33068430 PMC8664468

[39] Li D, Y XJ, Z CM. Research progress on the epidemiology and pathogenesis of severe fever with thrombocytopenia syndrome virus. Mod Preventative Med. 2025;52:961–6+76.

[40] Kim KH, Kim A, Noh M, et al. Seroprevalence and epidemiological insights into severe fever with thrombocytopenia syndrome on Jeju island, Republic of Korea. Viruses. 2025;17(4):17. doi: 10.3390/v17040466 PMC12031512 40284909

[41] Lizhao D. Analysis of clinical characteristics and death factors of fever with thrombocytopenia syndrome [chinese master’s thesis]. Nanjing Medical University; 2018. Available from: https://www.cnki.net/.

[42] He CQ, Ding NZ. Discovery of severe fever with thrombocytopenia syndrome bunyavirus strains originating from intragenic recombination. J Virol. 2012;86(22):12426–12430. doi: 10.1128/JVI.01317-12 22933273 PMC3486477

[43] Ma W, Hao Y, Peng C, et al. Analysis of gene differences between F and B epidemic lineages of Bandavirus Dabieense. Microorganisms. 2025;13(2):292. doi: 10.3390/microorganisms13020292 40005658 PMC11857831

[44] Lam TT, Liu W, Bowden TA, et al. Evolutionary and molecular analysis of the emergent severe fever with thrombocytopenia syndrome virus. Epidemics. 2013;5(1):1–10. doi: 10.1016/j.epidem.2012.09.002 23438426 PMC4330987

[45] Huang X, Liu L, Du Y, et al. The evolutionary history and spatiotemporal dynamics of the fever, thrombocytopenia and leukocytopenia syndrome virus (FTLSV) in China. PLOS Negl Trop Dis. 2014;8(10):e3237. doi: 10.1371/journal.pntd.0003237 25329580 PMC4199521

[46] Yun MR, Ryou J, Choi W, et al. Genetic diversity and evolutionary history of Korean isolates of severe fever with thrombocytopenia syndrome virus from 2013–2016. Arch Virol. 2020;165(11):2599–2603. doi: 10.1007/s00705-020-04733-0 32699980 PMC7547961

[47] Liu B, He T, Wang C, et al. Establishment of a genotyping criteria for Bandavirus dabieense and confirmation of new genotypes. Sci Rep. 2025;15(1):11269. doi: 10.1038/s41598-025-94203-x 40175408 PMC11965412

[48] Yun SM, Park SJ, Park SW, et al. Molecular genomic characterization of tick- and human-derived severe fever with thrombocytopenia syndrome virus isolates from South Korea. PLOS Negl Trop Dis. 2017;11(9):e0005893. doi: 10.1371/journal.pntd.0005893 28937979 PMC5627960

[49] Yun SM, Park SJ, Kim YI, et al. Genetic and pathogenic diversity of severe fever with thrombocytopenia syndrome virus (SFTSV) in South Korea. JCI Insight. 2020;5(2). doi: 10.1172/jci.insight.129531 PMC7098714 31877113

[50] Pérez LJ, Baele G, Hong SL, et al. Ecological changes exacerbating the spread of invasive ticks has driven the dispersal of severe fever with thrombocytopenia syndrome virus throughout Southeast Asia. Mol Biol Evol. 2024;41(8):41. doi: 10.1093/molbev/msae173 PMC11349436 39191515

[51] Dai ZN, Peng XF, Li JC, et al. Effect of genomic variations in severe fever with thrombocytopenia syndrome virus on the disease lethality. Emerging Microbes & Infect. 2022;11:1672–1682. doi: 10.1080/22221751.2022.2081617 PMC9225783 35603493

[52] Tsuda Y, Igarashi M, Ito R, et al. The amino acid at position 624 in the glycoprotein of SFTSV (severe fever with thrombocytopenia virus) plays a critical role in low-pH-dependent cell fusion activity. Biomed Res. 2017;38(2):89–97. doi: 10.2220/biomedres.38.89 28442665

[53] Tani H, Kawachi K, Kimura M, et al. Identification of the amino acid residue important for fusion of severe fever with thrombocytopenia syndrome virus glycoprotein. Virology. 2019;535:102–110. doi: 10.1016/j.virol.2019.06.014 31299486

[54] Noda K, Tsuda Y, Kozawa F, et al. The polarity of an amino acid at position 1891 of severe fever with thrombocytopenia syndrome virus L protein is critical for the polymerase activity. Viruses. 2020;13(1):13. doi: 10.3390/v13010033 33375489 PMC7823514

[55] Choi Y, Park SJ, Sun Y, et al. Severe fever with thrombocytopenia syndrome phlebovirus non-structural protein activates TPL2 signalling pathway for viral immunopathogenesis. Nat Microbiol. 2019;4(3):429–437. doi: 10.1038/s41564-018-0329-x 30617349 PMC6548314

[56] Wu YX, Yang X, Leng Y, et al. Human-to-human transmission of severe fever with thrombocytopenia syndrome virus through potential ocular exposure to infectious blood. Int J Infect Dis. 2022;123:80–83. doi: 10.1016/j.ijid.2022.08.008 35987469

[57] Fang X, Hu J, Peng Z, et al. Epidemiological and clinical characteristics of severe fever with thrombocytopenia syndrome bunyavirus human-to-human transmission. PLOS Negl Trop Dis. 2021;15(4):e0009037. doi: 10.1371/journal.pntd.0009037 33930022 PMC8087050

[58] Ye C, Qi R. Risk factors for person-to-person transmission of severe fever with thrombocytopenia syndrome. Infect Control Hosp Epidemiol. 2021;42(5):582–585. doi: 10.1017/ice.2020.1258 33161921

[59] Kirino Y, Ishijima K, Miura M, et al. Seroprevalence of severe fever with thrombocytopenia syndrome virus in small-animal veterinarians and nurses in the Japanese prefecture with the highest case load. Viruses. 2021;13(2):229. doi: 10.3390/v13020229 33540629 PMC7912989

[60] Oshima H, Okumura H, Maeda K, et al. A patient with severe fever with thrombocytopenia syndrome (SFTS) infected from a sick dog with SFTS virus infection. Jpn J Infect Dis. 2022;75(4):423–426. doi: 10.7883/yoken.JJID.2021.796 35228501

[61] Li J, Wang C, Li X, et al. Direct transmission of severe fever with thrombocytopenia syndrome virus from farm-raised fur animals to workers in Weihai, China. Virol J. 2024;21(1):113. doi: 10.1186/s12985-024-02387-x 38760812 PMC11100147

[62] Jaeyoung M, Hyeokjin L, Ji-Hoon J, et al. Aerosol transmission of severe fever with thrombocytopenia syndrome virus during resuscitation - ERRATUM. Infect Control Hosp Epidemiol. 2019;40:620. 31084678 10.1017/ice.2019.83

[63] Gong Z, Gu S, Zhang Y, et al. Probable aerosol transmission of severe fever with thrombocytopenia syndrome virus in southeastern China. Clin Microbiol Infect. 2015;21(12):1115–1120. doi: 10.1016/j.cmi.2015.07.024 26255811

[64] Koga S, Takazono T, Ando T, et al. Severe fever with thrombocytopenia syndrome virus RNA in semen, Japan. Emerg Infect Dis. 2019;25(11):2127–2128. doi: 10.3201/eid2511.190061 31625854 PMC6810197

[65] Yoo JR, Choi JH, Kim YR, et al. Occupational risk of severe fever with thrombocytopenia syndrome in healthcare workers. Open Forum Infect Dis. 2019;6(5):ofz210. doi: 10.1093/ofid/ofz210 31139678 PMC6527088

[66] Guardado-Calvo P, Rey FA. The envelope proteins of the bunyavirales. Adv Virus Res. 2017;98:83–118. 28433053 10.1016/bs.aivir.2017.02.002

[67] Hofmann H, Li X, Zhang X, et al. Severe fever with thrombocytopenia virus glycoproteins are targeted by neutralizing antibodies and can use DC-SIGN as a receptor for pH-dependent entry into human and animal cell lines. J Virol. 2013;87(8):4384–4394. doi: 10.1128/JVI.02628-12 23388721 PMC3624395

[68] Liu J, Xu M, Tang B, et al. Single-particle tracking reveals the sequential entry process of the bunyavirus severe fever with thrombocytopenia syndrome virus. Small. 2019;15(6):e1803788. doi: 10.1002/smll.201803788 30589216

[69] Yuan F, Zheng A. Entry of severe fever with thrombocytopenia syndrome virus. Virol Sin. 2017;32(1):44–50. doi: 10.1007/s12250-016-3858-6 27995422 PMC6598886

[70] Zhang L, Peng X, Wang Q, et al. CCR2 is a host entry receptor for severe fever with thrombocytopenia syndrome virus. Sci Adv. 2023;9(31):eadg6856. doi: 10.1126/sciadv.adg6856 37531422 PMC10396298

[71] Liu T, Li J, Liu Y, et al. SNX11 identified as an essential host factor for SFTS virus infection by CRISPR knockout screening. Virol Sin. 2019;34(5):508–520. doi: 10.1007/s12250-019-00141-0 31215001 PMC6814687

[72] Drake MJ, Brennan B, Briley K, et al. A role for glycolipid biosynthesis in severe fever with thrombocytopenia syndrome virus entry. PLOS Pathog. 2017;13(4):e1006316. doi: 10.1371/journal.ppat.1006316 28388693 PMC5397019

[73] Yamaoka S, Weisend C, Ebihara H. Identifying target cells for a tick-borne virus that causes fatal hemorrhagic fever. J Clin Invest. 2020;130(2):598–600. doi: 10.1172/JCI134512 31904585 PMC6994110

[74] Wang YN, Zhang YF, Peng XF, et al. Mast cell-derived proteases induce endothelial permeability and vascular damage in severe fever with thrombocytopenia syndrome. Microbiol Spectr. 2022;10(3):e0129422. doi: 10.1128/spectrum.01294-22 35612327 PMC9241724

[75] Li XK, Zhang SF, Xu W, et al. Vascular endothelial injury in severe fever with thrombocytopenia syndrome caused by the novel bunyavirus. Virology. 2018;520:11–20. doi: 10.1016/j.virol.2018.05.001 29754008

[76] Fang L, Yu S, Tian X, et al. Severe fever with thrombocytopenia syndrome virus replicates in platelets and enhances platelet activation. J Thromb Haemost. 2023;21(5):1336–1351. doi: 10.1016/j.jtha.2023.02.006 36792011

[77] Qu B, Qi X, Wu X, et al. Suppression of the interferon and NF-κB responses by severe fever with thrombocytopenia syndrome virus. J Virol. 2012;86(16):8388–8401. doi: 10.1128/JVI.00612-12 22623799 PMC3421730

[78] Liu Y, Wu B, Paessler S, et al. The pathogenesis of severe fever with thrombocytopenia syndrome virus infection in alpha/beta interferon knockout mice: insights into the pathologic mechanisms of a new viral hemorrhagic fever. J Virol. 2014;88(3):1781–1786. doi: 10.1128/JVI.02277-13 24257618 PMC3911604

[79] Suzuki T, Sato Y, Sano K, et al. Severe fever with thrombocytopenia syndrome virus targets B cells in lethal human infections. J Clin Invest. 2020;130(2):799–812. doi: 10.1172/JCI129171 31904586 PMC6994144

[80] Matsuno K, Orba Y, Maede-White K, et al. Animal models of emerging tick-borne phleboviruses: determining target cells in a lethal model of SFTSV infection. Front Microbiol. 2017;8:104. doi: 10.3389/fmicb.2017.00104 28194148 PMC5276813

[81] Wang T, Xu L, Zhu B, et al. Immune escape mechanisms of severe fever with thrombocytopenia syndrome virus. Front Immunol. 2022;13:937684. doi: 10.3389/fimmu.2022.937684 35967309 PMC9366518

[82] Jin C, Liang M, Ning J, et al. Pathogenesis of emerging severe fever with thrombocytopenia syndrome virus in C57/BL6 mouse model. Proc Natl Acad Sci U S A. 2012;109:10053–10058. 22665769 10.1073/pnas.1120246109 PMC3382536

[83] Zhang L, Fu Y, Wang H, et al. Severe fever with thrombocytopenia syndrome virus-induced macrophage differentiation is regulated by miR-146. Front Immunol. 2019;10:1095. doi: 10.3389/fimmu.2019.01095 31156641 PMC6529556

[84] Xing B. Study on the epidemiological and clinical features of severe fever with thrombocytopenia syndrome [chinese master’s thesis]. Academy of Military Sciences. 2018. Available from: https://www.cnki.net/

[85] Tang X, Wu W, Wang H, et al. Human-to-human transmission of severe fever with thrombocytopenia syndrome bunyavirus through contact with infectious blood. J Infect Dis. 2013;207(5):736–739. doi: 10.1093/infdis/jis748 23225899

[86] Li J, Li S, Yang L, et al. Severe fever with thrombocytopenia syndrome virus: a highly lethal bunyavirus. Crit Rev Microbiol. 2021;47(1):112–125. doi: 10.1080/1040841X.2020.1847037 33245676

[87] Huang M, Wang T, Huang Y, et al. The clinical and immunological characteristics in fatal severe fever with thrombocytopenia syndrome virus (SFTSV) infection. Clin Immunol (orlando, Fla). 2023;248:109262. doi: 10.1016/j.clim.2023.109262 36796470

[88] Liu S, Chai C, Wang C, et al. Systematic review of severe fever with thrombocytopenia syndrome: virology, epidemiology, and clinical characteristics. Rev Med Virol. 2014;24(2):90–102. doi: 10.1002/rmv.1776 24310908 PMC4237196

[89] Gai ZT, Zhang Y, Liang MF, et al. Clinical progress and risk factors for death in severe fever with thrombocytopenia syndrome patients. J Infect Dis. 2012;206(7):1095–1102. doi: 10.1093/infdis/jis472 22850122

[90] Park SY, Kwon JS, Kim JY, et al. Severe fever with thrombocytopenia syndrome-associated encephalopathy/encephalitis. Clin Microbiol Infect. 2018;24(4):.e432.1–.e.4. doi: 10.1016/j.cmi.2017.09.002 28899841

[91] Wang M, Huang P, Liu W, et al. Risk factors of severe fever with thrombocytopenia syndrome combined with central neurological complications: a five-year retrospective case-control study. Front Microbiol. 2022;13:1033946. doi: 10.3389/fmicb.2022.1033946 36406394 PMC9668900

[92] Cui N, Bao XL, Yang ZD, et al. Clinical progression and predictors of death in patients with severe fever with thrombocytopenia syndrome in China. J Clin Virol. 2014;59(1):12–17. doi: 10.1016/j.jcv.2013.10.024 24257109

[93] Hou H, Zou S, Wei W, et al. Kinetics and prognostic significance of laboratory markers in patients with severe fever with thrombocytopenia syndrome: insight from a comprehensive analysis. J Infect Dis. 2024;229(6):1845–1855. doi: 10.1093/infdis/jiad426 37804100

[94] Li H, Lu QB, Xing B, et al. Epidemiological and clinical features of laboratory-diagnosed severe fever with thrombocytopenia syndrome in China, 2011–17: a prospective observational study. Lancet Infect Dis. 2018;18(10):1127–1137. doi: 10.1016/S1473-3099(18)30293-7 30054190

[95] Yang K, Chen J, Chen Z, et al. Risk factors for death in patients with severe fever with thrombocytopenia syndrome. Am J Trop Med Hyg. 2023;109(1):94–100. doi: 10.4269/ajtmh.22-0667 37253446 PMC10324000

[96] Chen X, Yu Z, Qian Y, et al. Clinical features of fatal severe fever with thrombocytopenia syndrome that is complicated by invasive pulmonary aspergillosis. J Infect Chemother. 2018;24(6):422–427. doi: 10.1016/j.jiac.2018.01.005 29428567

[97] Hiraki T, Yoshimitsu M, Suzuki T, et al. Two autopsy cases of severe fever with thrombocytopenia syndrome (SFTS) in Japan: a pathognomonic histological feature and unique complication of SFTS. Pathol Int. 2014;64(11):569–575. doi: 10.1111/pin.12207 25329676 PMC4282027

[98] Kawaguchi T, Matsuda M, Takajo I, et al. Severe fever with thrombocytopenia syndrome with myocardial dysfunction and encephalopathy: a case report. J Infect Chemother. 2016;22(9):633–637. doi: 10.1016/j.jiac.2016.01.022 26943978

[99] National Health and Family Planning Commission of the People’s Replublic China. Guide for severe fever with thrombocytopenia syndrome control. 2010 (edit). [cited 2013 Sept 23]. Available from: http://www.moh.gov.cn/mohwsyjbgs/s8348/201010/49272.shtml (in Chinese).

[100] Yoshikawa T, Fukushi S, Tani H, et al. Sensitive and specific PCR systems for detection of both Chinese and Japanese severe fever with thrombocytopenia syndrome virus strains and prediction of patient survival based on viral load. J Clin Microbiol. 2014;52(9):3325–3333. doi: 10.1128/JCM.00742-14 24989600 PMC4313158

[101] Zhou J, Wang Q, Zhu L, et al. Development and evaluation of a rapid detection assay for severe fever with thrombocytopenia syndrome virus based on reverse-transcription recombinase polymerase amplification. Mol Cell Probes. 2020;52:101580. doi: 10.1016/j.mcp.2020.101580 32330556 PMC7172814

[102] Huang XY, Hu XN, Ma H, et al. Detection of new bunyavirus RNA by reverse transcription-loop-mediated isothermal amplification. J Clin Microbiol. 2014;52(2):531–535. doi: 10.1128/JCM.01813-13 24478484 PMC3911317

[103] Baek YH, Cheon HS, Park SJ, et al. Simple, rapid and sensitive portable molecular diagnosis of SFTS virus using reverse transcriptional loop-mediated isothermal amplification (RT-LAMP). J Microbiol Biotechnol. 2018;28(11):1928–1936. doi: 10.4014/jmb.1806.06016 30270605

[104] Zhang Y, Bai X, Li J, et al. A CRISPR-based nucleic acid detection method for severe fever with thrombocytopenia syndrome virus. Virus Res. 2022;311:198691. doi: 10.1016/j.virusres.2022.198691 35143909

[105] Ningning C, Yanhua D, Xueyong H, et al. Comparison and evaluation of different assays in the diagnosis of severe fever with thrombocytopenia syndrome. Tianjin Med J. 2017;45(2):210–214.

[106] Jiao Y, Zeng X, Guo X, et al. Preparation and evaluation of recombinant severe fever with thrombocytopenia syndrome virus nucleocapsid protein for detection of total antibodies in human and animal sera by double-antigen sandwich enzyme-linked immunosorbent assay. J Clin Microbiol. 2012;50(2):372–377. doi: 10.1128/JCM.01319-11 22135253 PMC3264160

[107] Zhan J, Wang Q, Cheng J, et al. Current status of severe fever with thrombocytopenia syndrome in China. Virol Sin. 2017;32(1):51–62. doi: 10.1007/s12250-016-3931-1 28251515 PMC6598917

[108] Kim DM, Yu BJ, Kim DY, et al. Clinically differential diagnosis of human granulocytic anaplasmosis and severe fever with thrombocytopenia syndrome. Sci Rep. 2023;13(1):6837. doi: 10.1038/s41598-023-32061-1 37100782 PMC10133271

[109] Qi R, Qin XR, Wang L, et al. Severe fever with thrombocytopenia syndrome can masquerade as hemorrhagic fever with renal syndrome. PLOS Negl Trop Dis. 2019;13(3):e0007308. doi: 10.1371/journal.pntd.0007308 30925154 PMC6457554

[110] Malavige GN, Wijewickrama A, Ogg GS. Differentiating dengue from other febrile illnesses: a dilemma faced by clinicians in dengue endemic countries. Lancet Global Health. 2023;11(3):e306–e307. doi: 10.1016/S2214-109X(22)00547-2 36796966

[111] Yu-Xian X, Dongxia L, Jie F. Advances in diagnosis and treatment of Lyme disease. World Notes Antibiot. 2022;43(1):10.

[112] Le Van N, Pham Van C, Nguyen Dang M, et al. Clinical features, laboratory characteristics and prognostic factors of severity in patients with rickettsiaceae at two military hospitals, Northern Vietnam. Infect Drug Resist. 2020;13:2129–2138. doi: 10.2147/IDR.S253540 32753908 PMC7351622

[113] Shahhosseini N, Wong G, Babuadze G, et al. Crimean-Congo hemorrhagic fever virus in Asia, Africa and Europe. Microorganisms. 2021;9(9):1907. doi: 10.3390/microorganisms9091907 34576803 PMC8471816

[114] Guo WP, Xie GC, Li D, et al. Molecular detection and genetic characteristics of Babesia gibsoni in dogs in Shaanxi Province, China. Parasites & Vectors. 2020;13(1):366. doi: 10.1186/s13071-020-04232-w 32698848 PMC7376908

[115] Kotepui M, Kotepui KU, De Jesus Milanez G, et al. Plasmodium spp. mixed infection leading to severe malaria: a systematic review and meta-analysis. Sci Rep. 2020;10(1):11068. doi: 10.1038/s41598-020-68082-3 32632180 PMC7338391

[116] Wu L, van den Hoogen LL, Slater H, et al. Comparison of diagnostics for the detection of asymptomatic Plasmodium falciparum infections to inform control and elimination strategies. Nature. 2015;528(7580):S86–93. doi: 10.1038/nature16039 26633770

[117] Jinghua C. Diagnosis and prevention measures of porcine leptospirosis. Chin J Anim Husb Vet Med. 2023;2:136–138.

[118] Yu KM, Park SJ, Yu MA, et al. Cross-genotype protection of live-attenuated vaccine candidate for severe fever with thrombocytopenia syndrome virus in a ferret model. Proc Natl Acad Sci U S A. 2019;116:26900–26908. 31818942 10.1073/pnas.1914704116 PMC6936527

[119] Dong F, Li D, Wen D, et al. Single dose of a rVSV-based vaccine elicits complete protection against severe fever with thrombocytopenia syndrome virus. NPJ Vaccines. 2019;4(1):5. doi: 10.1038/s41541-018-0096-y 30701094 PMC6347601

[120] Gary EN, Weiner DB. DNA vaccines: prime time is now. Curr Opin Immunol. 2020;65:21–27. doi: 10.1016/j.coi.2020.01.006 32259744 PMC7195337

[121] Kang JG, Jeon K, Choi H, et al. Vaccination with single plasmid DNA encoding IL-12 and antigens of severe fever with thrombocytopenia syndrome virus elicits complete protection in IFNAR knockout mice. PLOS Negl Trop Dis. 2020;14(3):e0007813. doi: 10.1371/journal.pntd.0007813 32196487 PMC7112229

[122] Sabbaghi A, Miri SM, Keshavarz M, et al. Inactivation methods for whole influenza vaccine production. Rev Med Virol. 2019;29(6):e2074. doi: 10.1002/rmv.2074 31334909

[123] Aqian L. Immunogenicity and protective efficacy of an inactivated SFTS vaccine candidate in mice. 2022.

[124] Benelli G, Pavela R, Canale A, et al. Tick repellents and acaricides of botanical origin: a green roadmap to control tick-borne diseases? Parasitol Res. 2016;115(7):2545–2560. doi: 10.1007/s00436-016-5095-1 27146901

[125] Piesman J, Eisen L. Prevention of tick-borne diseases. Annu Rev Entomol. 2008;53(1):323–343. doi: 10.1146/annurev.ento.53.103106.093429 17877457

[126] Jia N, Wang J, Shi W, et al. Large-scale comparative analyses of tick genomes elucidate their genetic diversity and vector capacities. Cell. 2020;182(5):1328–1340.e13. doi: 10.1016/j.cell.2020.07.023 32814014

[127] Sidwell RW, Robins RK, Hillyard IW. Ribavirin: an antiviral agent. Pharmacol & Ther. 1979;6:123–146. doi: 10.1016/0163-7258(79)90058-5 390559

[128] Liu W, Lu QB, Cui N, et al. Case-fatality ratio and effectiveness of ribavirin therapy among hospitalized patients in China who had severe fever with thrombocytopenia syndrome. Clin Infect Dis. 2013;57(9):1292–1299. doi: 10.1093/cid/cit530 23965284

[129] Xia G, Sun S, Zhou S, et al. A new model for predicting the outcome and effectiveness of drug therapy in patients with severe fever with thrombocytopenia syndrome: a multicenter Chinese study. PLOS Negl Trop Dis. 2023;17(3):e0011158. doi: 10.1371/journal.pntd.0011158 36877734 PMC10019728

[130] MediVector, Inc. A phase 3, randomized, double-blind, placebo-controlled, multicenter study evaluating the efficacy and safety of favipiravir in adult subjects with uncomplicated influenza. 2016. Available from: https://www.clinicaltrials.gov./ct2/show/NCT02008344?term=favipiravir&rank=3

[131] Tani H, Fukuma A, Fukushi S, et al. Efficacy of T-705 (Favipiravir) in the treatment of infections with lethal severe fever with thrombocytopenia syndrome virus. mSphere. 2016;1(1):1. doi: 10.1128/mSphere.00061-15 PMC4863605 27303697

[132] Li H, Jiang XM, Cui N, et al. Clinical effect and antiviral mechanism of T-705 in treating severe fever with thrombocytopenia syndrome. Sig Transduct Target Ther. 2021;6(1):145. doi: 10.1038/s41392-021-00541-3 PMC8050330 33859168

[133] Sakurai Y, Kolokoltsov AA, Chen CC, et al. Ebola virus. Two-pore channels control Ebola virus host cell entry and are drug targets for disease treatment. Sci (New York, NY). 2015;347(6225):995–998. doi: 10.1126/science.1258758 PMC4550587 25722412

[134] DeWald LE, Dyall J, Sword JM, et al. The calcium channel blocker bepridil demonstrates efficacy in the murine model of marburg virus disease. J Infect Dis. 2018;218(suppl_5):SS588–sS591. doi: 10.1093/infdis/jiy332 PMC6249584 29982632

[135] Scherbik SV, Brinton MA. Virus-induced Ca2+ influx extends survival of west Nile virus-infected cells. J Virol. 2010;84(17):8721–8731. doi: 10.1128/JVI.00144-10 20538858 PMC2918993

[136] Li H, Zhang LK, Li SF, et al. Calcium channel blockers reduce severe fever with thrombocytopenia syndrome virus (SFTSV) related fatality. Cell Res. 2019;29(9):739–753. doi: 10.1038/s41422-019-0214-z 31444469 PMC6796935

[137] Liu S, Liu H, Zhang K, et al. Proteasome inhibitor PS-341 effectively blocks infection by the severe fever with thrombocytopenia syndrome virus. Virol Sin. 2019;34(5):572–582. doi: 10.1007/s12250-019-00162-9 31637631 PMC6814677

[138] Baba M, Okamoto M, Toyama M, et al. Amodiaquine derivatives as inhibitors of severe fever with thrombocytopenia syndrome virus (SFTSV) replication. Antiviral Res. 2023;210:105479. doi: 10.1016/j.antiviral.2022.105479 36566117

[139] Shen S, Zhang Y, Yin Z, et al. Antiviral activity and mechanism of the antifungal drug, anidulafungin, suggesting its potential to promote treatment of viral diseases. BMC Med. 2022;20(1):359. doi: 10.1186/s12916-022-02558-z 36266654 PMC9585728

[140] Ogawa M, Shirasago Y, Ando S, et al. Caffeic acid, a coffee-related organic acid, inhibits infection by severe fever with thrombocytopenia syndrome virus in vitro. J Infect Chemother. 2018;24(8):597–601. doi: 10.1016/j.jiac.2018.03.005 29628386

[141] Chen L, Chen T, Li R, et al. Recent advances in the study of the immune escape mechanism of SFTSV and Its therapeutic agents. Viruses. 2023;15(4):15. doi: 10.3390/v15040940 PMC10142331 37112920

[142] Huang X, Ding S, Jiang X, et al. Detection of SFTS virus RNA and antibodies in severe fever with thrombocytopenia syndrome surveillance cases in endemic areas of China. BMC Infect Dis. 2019;19(1):476. doi: 10.1186/s12879-019-4068-2 31138131 PMC6540372

[143] Yoshikawa T. Vaccine development for severe fever with thrombocytopenia syndrome. Viruses. 2021;13(4):627. doi: 10.3390/v13040627 33917632 PMC8067456

